# More than mesolectic: Characterizing the nutritional niche of *Osmia cornifrons*

**DOI:** 10.1002/ece3.10640

**Published:** 2023-10-20

**Authors:** Makaylee K. Crone, Natalie K. Boyle, Sean T. Bresnahan, David J. Biddinger, Rodney T. Richardson, Christina M. Grozinger

**Affiliations:** ^1^ Department of Entomology, Center for Pollinator Research, Huck Institutes of the Life Sciences Pennsylvania State University University Park Pennsylvania USA; ^2^ Intercollege Graduate Program in Ecology, Huck Institutes of the Life Sciences Pennsylvania State University University Park Pennsylvania USA; ^3^ Intercollege Graduate Degree Program in Molecular, Cellular, and Integrative Biosciences, Huck Institutes of the Life Sciences Pennsylvania State University University Park Pennsylvania USA; ^4^ Penn State Fruit Research and Extension Center Biglerville Pennsylvania USA; ^5^ University of Maryland Center for Environmental Sciences College Park Maryland USA

**Keywords:** DNA metabarcoding, nutrition, pollen, wild bees

## Abstract

Characterizing the nutritional needs of wild bee species is an essential step to better understanding bee biology and providing suitable supplemental forage for at‐risk species. Here, we aim to characterize the nutritional needs of a model solitary bee species, *Osmia cornifrons* (Radoszkowski), by using dietary protein‐to‐lipid ratio (P:L ratio) as a proxy for nutritional niche and niche breadth. We first identified the mean target P:L ratio (~3.02:1) and P:L collection range (0.75–6.26:1) from pollen provisions collected across a variety of sites and time points. We then investigated the P:L tolerance range of larvae by rearing bees in vitro on a variety of diets. Multifloral and single‐source pollen diets with P:L ratios within the range of surveyed provisions did not always support larval development, indicating that other dietary components such as plant secondary compounds and micronutrients must also be considered in bee nutritional experiments. Finally, we used pollen metabarcoding to identify pollen from whole larval provisions to understand how much pollen bees used from plants outside of their host plant families to meet their nutritional needs, as well as pollen from individual forager bouts, to observe if bees maintained strict floral constancy or visited multiple plant genera per foraging bout. Whole larval provision surveys revealed a surprising range of host plant pollen use, ranging from ~5% to 70% of host plant pollen per provision. Samples from individual foraging trips contained pollen from multiple genera, suggesting that bees are using some form of foraging decision making. Overall, these results suggest that *O. cornifrons* have a wide nutritional niche breadth, but while pollen P:L ratio tolerance is broad, a tolerable P:L ratio alone is not enough to create a quality diet for *O. cornifrons*, and the plant species that make up these diets must also be carefully considered.

## Graphical abstract

*Osmia cornifrons* compose larval pollen provisions with a wide range in P:L ratio, ranging from 0.75 to 6.26:1. In vitro studies found that larval survival did not depend on dietary pollen P:L ratio, indicating that other factors, such as micronutrients and plant secondary compounds, could be responsible for changes in larval survival. Bees also collected pollen from many plant genera, both within and outside of their host plant families. We conclude that *O. cornifrons* have a wide nutritional niche breadth, but that balancing different plant genera may be important to limit toxic secondary compounds and ensure proper micronutrient concentrations.

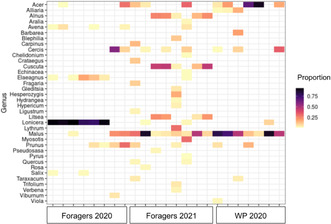

## 1 INTRODUCTION

Wild bee diversity and abundance are in decline worldwide (Zattara & Aizen, 2021). One of the greatest stressors that contributes to this decline is a lack of quality nutritional resources (LeBuhn & Vargas Luna, 2021), and understanding the nutritional needs of wild bees is paramount to creating planting schemes to improve bee habitat (Crone et al., 2022). Bees collect pollen (which is their primary source of protein and lipids) and nectar (their primary source of carbohydrates) to feed their larvae (Leach & Drummond, 2018), and the nutritional profiles of these resources are shaped evolutionarily by plant‐bee interactions (Ruedenauer et al., 2019).

A bee species' dietary preferences are composed of (1) a nutritional target, or an ideal ratio of nutrients (e.g. protein: lipids, protein: carbohydrates) that they must maintain for optimal fitness; (2) the niche breadth surrounding that ratio, or the tolerance range of a species outside of that ratio; and (3) the foraging behavior a species utilizes to meet their nutritional needs (Parreño et al., 2022). Bee species can range from strict foraging specialists (that visit only one plant species) to broad foraging generalists (that visit plants from many families; Michener, 2007), and there have been substantial efforts to document these relationships in plant‐pollinator interaction networks for many species (Bloom et al., 2022; Burkle et al., 2013; Vizentin‐Bugoni et al., 2018). The nutritional requirements, or target, of pollen‐derived macronutrients (proteins and lipids, or the P:L ratio) are also thought to vary across bee species (Vaudo, Tooker, et al., 2020), and bumble bees (Bombus impatiens) foraging patterns have previously been explained by plant pollen P:L ratios (Vaudo et al., 2016). However, niche breadth across bee species has been traditionally understudied (Parreño et al., 2022), and studies investigating how foraging behavior is related to nutritional balancing have not been conducted in species outside the genus Bombus. Species may be broadly categorized as macronutrient generalists (able to tolerate wide variation in macronutrients, and corresponding nutritional niche space) or macronutrient specialists (able to tolerate only a narrow range in macronutrients, and nutritional niche space), but hard cutoff points or rules for macronutrient generalists and specialists are poorly defined (Machovsky‐Capuska et al., 2016). We suggest that to categorize a bee species as a macronutrient specialist, it must be demonstrated that variation in the P:L ratio of pollen collected is narrower intra‐specifically than it is inter‐specifically (and vice versa for generalists). However, a lack of studies concerning macronutrient variation across bees, and pollen‐consuming insects in general (Behmer & Joern, 2008), makes comparing and classifying species as generalist, specialist, or somewhere in‐between, challenging. Thus, characterizing the nutritional niches of a diversity of bee species is a necessary first step in understanding how different bee species partition floral resources and fit within this framework.

Understanding bee foraging behavior can also lend insight to bee nutritional niche space partitioning. For example, determining if female bees collect pollen from many plant genera during a single foraging bout (suggesting they may balance pollen collections as they forage) or across foraging bouts (balancing pollen collections over longer time periods) can provide evidence as to how bees are making foraging decisions (Eckhardt et al., 2014; Smith et al., 2019; Williams & Tepedino, 2003). Understanding bee nutritional niches and foraging decision making can lead to more detailed studies, such as how bees perceive risks and rewards, and provides insight as to which planting configurations of floral resources are most useful when designing habitat planting schemes for wild bees. Such studies on risk–reward (Fülöp & Menzel, 2000; Jones & Dornhaus, 2011; Tan et al., 2015) have been extensively studied in social bee species, such as *Bombus* sp. and *Apis melifera*, but are lacking in solitary species, or bulk, pollen protein and lipid ratios have previously been used to describe and compare bee nutritional preferences (Barraud et al., 2022; Crone & Grozinger, 2021; Vaudo et al., 2016, Vaudo, Tooker, et al., 2020). Though pollen is also composed of a wide array of micronutrients, classes of lipids, and amino acids, this method can still demonstrate broad trends in nutritional differences across bee species (Vaudo, Tooker, et al., 2020). However, it should be noted that these methods can also co‐extract other non‐polar compounds from pollen (e.g. flavonoids, triacylglycerols), leading to an overestimation of total lipid concentration (Lau et al., 2022). Studies using these broad bulk extraction methods, while lacking specificity, can nonetheless lay the groundwork for future studies that assess bee nutritional needs in more detail. *cornifrons* (Radoszkowski; Hymenoptera: Megachilidae) is a univoltine, stem‐nesting, mass‐provisioning, early spring, solitary bee species that represents a unique model system with which to characterize a bee species' nutritional niche breadth. *O. cornifrons* has previously been characterized as a mesolectic species that collects pollen from plants primarily within the families Rosaceae and Fabaceae when provisioning nests for their offspring (Haider et al., 2014; Nagamitsu et al., 2018; Russo & Danforth, 2017). Recent studies using pollen metabarcoding of whole pollen provisions have suggested that though they primarily collect pollen from these plants (>70% of a pollen provision), populations in the North‐eastern United States also collect pollen from plants outside of these families (Vaudo, Biddinger, et al., 2020). *O. cornifrons* co‐evolved with Rosaceae and Fabaceae in their native range, Japan, but as introduced populations persist on plants that they did not co‐evolve with (both within and outside of these host families), this indicates an ability to adapt to new plant communities (Vaudo, Biddinger, et al., 2020). *O. cornifrons* macronutrient nutritional niche breadth falls between these bounds. Vaudo, Tooker, et al. (2020) also sampled 10 total *O. cornifrons* larval pollen provisions from 4 sites and determined the mean nutritional target for protein‐to‐lipid ratio (P:L ratio) was 2.9:1, which differed from pollen collected by generalist honey bees (*Apis mellifera* L., 1:1–2:1) and bumble bees (*Bombus impatiens* Cresson, ~4:1) collected from other locations. This study presents intriguing differences in nutritional P:L ratio targets between these species, but more complete characterization of the macronutrient nutritional niche of *O. cornifrons* requires examination of a larger sample size of provisions across sites and time periods to determine if there are changes in mean target nutritional ratio and find the true macronutrient niche breadth.

The aim of the present study is to further characterize the nutritional niche of *O. cornifrons* with greater statistical power. Our goal was to (1) determine the macronutrient P:L ratio nutritional target (i.e. the mean of collections across individuals), (2) determine the macronutrient niche breadth (i.e. P:L ratio tolerance range), and (3) describe trends in bee foraging behavior. More specifically, we wanted to observe if female bees collect pollen from many plant genera during a single foraging bout (balancing pollen collections as they forage) or across foraging bouts (balancing pollen collections over longer time periods). In doing so, our work improves on past efforts, since whole pollen provisions have been examined for species composition (Vaudo, Biddinger, et al., 2020) and pollen loads from individual forager trips have not. We first collected whole pollen provisions from *O. cornifrons* nests to determine the pollen P:L ratio nutritional target and the breadth, or range, of P:L ratios found across foraging individuals. We also assessed if pollen P:L ratios were consistent across space and time, which would provide evidence if bees consistently arrived at the same target dietary ratio as in Vaudo, Tooker, et al. (2020), regardless of variation in plant communities, either by selective foraging or by consistently visiting plants they have coevolved with. We then determined the relative proportions of each plant genera in individual foraging trip collections and whole pollen provisions by using pollen metabarcoding. Finally, we raised *O. cornifrons* from egg to adult on multifloral pollen diets that were artificially modified to have different P:L ratios, multifloral pollen diets with naturally variable P:L ratios, and pollens from single plant species with different P:L ratios to gain further insight on dietary tolerance ranges. These studies tracked both survival and larval development time to determine diet suitability, as in Eckhardt et al. (2014), and allowed us to determine if a P:L ratio in the range identified from pollen provisions in *O. cornifrons* nests was sufficient alone to provide a quality diet for larval development. As there are many variables that differ across pollen from different plant species (e.g. micronutrients, amino acids, etc.), this method was used to tease apart the effects of crude P:L ratio from these variables.

We hypothesized that whole pollen provisions from different sites and collection periods would have a mean target P:L ratio of 2.9:1 (similar to the prior preliminary sampling by Vaudo, Tooker, et al., 2020), and that P:L ratios would range from 1.6:1 (the mean of host plant family Fabaceae) to 3.8:1 (the mean of host plant family Rosaceae) due to their affinity to these plants (Vaudo, Tooker, et al., 2020). Similarly, we hypothesized that larvae fed pollen diets with P:L ratios outside of this range would have longer development times and a lower survival rate, which would indicate an ideal macronutrient niche breadth within this range. Finally, we expected that foragers would collect pollen from multiple plant genera during a single foraging trip (rather than maintaining strict floral constancy during a foraging trip), which could suggest that bees are balancing dietary P:L ratios within each foraging trip.

## 2 METHODS

### 2.1 Experiment group 1 – Examining *O. cornifrons* pollen collection composition

#### 2.1.1 Experiment 1A: Examining nutritional content of larval pollen provisions across sites and time

##### Pollen collection and analyses

Nutritional content variability was assessed both across sites (sites A–D) and across time points within a single site (site E). To examine variation across sites, unconsumed pollen balls from nesting tubes at trap nests were collected in September 2019 from pre‐established nesting populations after *O. cornifrons* nesting had ceased for the year. Trap nests were composed of a dark blue mail tote shelter, measuring 58.4 cm × 38.1 cm × 40.6 cm (Postal Products Unlimited), and made of weatherproof corrugated plastic sheets which housed approximately 1000 cardboard nesting tubes (152 mm long × 8 mm diameter). Mail totes were UV reflective and attractive to *Osmia* (Artz et al., 2014). Totes were fit with hardware cloth across the entrance to prevent predation by birds and rodents and propped on top of cinder blocks to allow for water drainage. Pollen provisions were sampled from four different nesting sites at the Penn State Fruit Research and Extension Center (Sites A–D, Biglerville, PA; Table S1). Sites A, C, and D were orchards or wooded sites while site B was in a nearby suburban area.

To examine nutritional variation over time at a single site, Site E, whole larval provision masses were collected weekly from trap nests from May 3rd to June 6th, 2019 (6 weeks). All completed nesting tubes were removed each week to prevent sampling from prior weeks. As a populating of egg‐laying females had not yet been established at this site, 500 mated adults were released at trap nests in 4 weekly releases in May of 2019 (2000 adults total, 60:40 Male:Female) throughout sampling.

Only one sample per nesting tube was collected in both sample sets to avoid autocorrelation among individual females, and samples were randomly collected from different sections of each tube to ensure minimal temporal bias and any bias resulting from male versus female pollen provisions. Samples were frozen at −20°C until analyses. Samples were lyophilized (FreeZone 6 Plus; Labconco) and pollen protein and lipid concentrations were measured using a modified protocol (using glass tubes in lipid analyses to prevent contamination from plastic residues) from Vaudo, Tooker, et al. (2020). Experimental protocols can be found in the Supporting Information.

##### Data analysis

A Shapiro‐Wilks test determined that the data were normally distributed. Thus, a fixed effects ANOVA and Tukey's HSD with a Bonferroni correction were used to determine if there were significant differences among weeks and sites sampled. Variables site and week (time point) were fixed effect predictor variables, and protein concentration, lipid concentration, and P:L ratio were outcome variables. There were no random effects or covariates.

#### 2.1.2 Experiment 1B: Examining the species composition and richness of whole pollen provisions and collections from individual foraging trips

##### Pollen collection and DNA metabarcoding

Whole larval pollen provisions and individual adult foragers with pollen visible on the scopa (*n* = 8 from 2020, *n* = 8 from 2021) were collected from a single site, the Pennsylvania State Game Lands #176 (Site F, Centre County, PA). Whole larval pollen provisions were collected from completed nesting tubes on May 13th, 2020 and frozen prior to analysis (*n = 7*). Bees were still actively provisioning nests at this site, which allowed us to randomize collections to include equal amounts of male and female pollen provisions (this was approximately determined by location within the nesting tube and provision size; Rau, 1937). Only one pollen provision per nesting tube was collected to include collections from a diversity of foragers. Adult foragers were collected by hand netting on May 21st and 26th in 2020 (*n* = 8) and May 1st and 6th in 2021 (*n* = 8). Foragers were only collected on sunny days above 12.7°C between 9 am and 3 pm. Only females with visible pollen loads on scopae were collected to ensure detected pollen was collected for larval provisions and not the result of pollen transfer during nectar foraging by adults. Bees and provision masses were frozen at −20°C until analysis.

Pollen was removed from bees by vortexing them individually for 30 s while submerged in 1% sodium dodecyl sulfate (SDS) and 2% polyvinyl pyrrolidinone (PVP) solution (w/w; Lucas et al., 2018). Bees were removed and tubes were centrifuged at 16,000 *g* to pellet pollen. This supernatant was discarded, and the pollen pellet proceeded to DNA extraction (see below). Whole pollen provisions were homogenized with a mortar and pestle, and 100 mg of each provision was sub‐sampled for DNA extraction. DNA metabarcoding was conducted by following protocols from Sponsler et al. (2020) with the nuclear ribosomal spacer regions ITS1 and ITS2 as targets. Briefly, we homogenized samples with 0.7 mm zirconium beads in a bead beater (Omni Bead Ruptor 24 Elite) using 4 1‐min intervals at 7 m/s with 1‐min ice bath incubations between intervals. DNA was then extracted by using the DNeasy Plant mini kit (Qaigen). Loci were then amplified through three rounds of PCR in a nested design. PCR 1 amplified target regions with generic primers, PCR 2 attached next‐generation sequencing read‐priming oligonucleotides, and PCR 3 added dual multiplex indices. Completed libraries were then sequenced using 2 × 300 Illumina MiSeq kits at the Penn State Genomics Core Facility, resulting in 301 bp paired‐end reads.

##### DNA metabarcoding bioinformatics pipeline

Barcode analysis was conducted with vsearch (Rognes et al., 2016). Paired‐end reads were merged with command –*fastq_mergepairs*. The command –*usearch_global* was used to compare query sequences to plant database target sequences using semi‐global alignment (‐‐gapopen 0TE ‐‐gapext 0TE) with a minimum pairwise identity of 95%, a minimum query coverage of 80%, and a maximum of 100 and a minimum of 50 accepted matches. As a quality control, a query match for at least 5% of reads per sample was required for both markers combined. Custom Python scripts were used to relate vsearch top‐hit alignments to taxonomic lineages and summarize the data into tables of sequence counts per taxon for both loci. All commands and code used to run this analysis are provided at https://github.com/RTRichar/SimpleSequenceClassification.

Following taxonomic annotation, genus level annotations were used for all remaining analysis. Samples with more than 5% of merged reads matching query sequences when ITS1 and ITS2 loci were combined were considered to pass the quality threshold (no samples removed). Samples and their matching genera were then compiled into one document for each locus and exported to R studio (R Core Team, 2021 version 4.1.3) for further processing. The package dplyr (Wickham et al., 2022) was used to group data with plant genera as rows and sequencing reads for ITS1 and ITS2 as columns for each sample. The maximum number of sequencing reads from ITS1 and ITS2 was determined for each plant genus by sample and divided by the total number of reads in that sample (the sum of the maximum read numbers) to obtain the proportional value. All plant genera with 1% or greater proportion of the reads matching plant sequences in a sample were included in the final analyses.

2.7% of ITS1 barcode sequences and 6.9% of ITS2 barcode sequences generated by MetaCurator were found to be sufficiently indistinct to assign sequencing reads to genera. We identified these sequences by using MetaCleaner (https://github.com/sbresnahan/metacleaner), a custom software for cleaning reference sequence databases generated by MetaCurator. All non‐plant ITS1 and ITS2 sequences were retrieved from NCBI with EFetch (Kans, 2023) implemented in the R package reutils (https://github.com/gschofl/reutils) using search parameters ((((ITS1) OR 5.8S) OR 28S) OR ITS2) NOT Embryophyta[Organism] AND (“0”[SLEN]: “10,000”[SLEN]) to construct a “non‐plant” blastn sequence database. Barcode sequences generated with MetaCurator were searched for hits with 100% sequence identity and 100% query coverage against the non‐plant sequence database; matching barcodes (corresponding to fungal sequences rather than the plant sequences they were misidentified as) were flagged as mislabeled. Then, all plant ITS1 and ITS2 sequences were retrieved from NCBI with Entrez using search parameters ((((ITS1) OR 5.8S) OR 28S) OR ITS2) AND Embryophyta[Organism] AND (“0”[SLEN]: “10,000”[SLEN]) to construct a “plant” blastn sequence database. The remaining barcodes were then searched for hits with 100% sequence identity and 100% query coverage against the plant sequence database; barcodes with matches were flagged as potentially correctly labeled, and those without matches were flagged as potentially mislabeled. Finally, taxonomy information for all potentially correctly labeled and potentially mislabeled barcode accessions and their matches to plant sequence accessions was retrieved using taxonomizr (https://github.com/sherrillmix/taxonomizr). For each barcode, taxonomy information was compared with that of the exact matches – potentially correctly labeled barcodes with exact matches to sequences of a different genus (than they were labeled as on NCBI) were flagged as mislabeled, while those with exact matches to sequences of the same genus were flagged as correctly labeled. Additionally, those initially flagged as potentially mislabeled (no hits against plant sequences at 100% sequence identity and 100% query coverage) but with the top hit being against a sequence of the same genus were flagged as correctly labeled. Finally, all barcodes with exact matches to sequences of organisms other than in the class Magnoliopsida were flagged as mislabeled. All barcodes flagged as mislabeled were then filtered from the sequence database.

##### Data analysis

Genus richness of whole pollen provisions and individual foraging trips were plotted as boxplots for visual comparison with base R (R Core Team, 2021 version 4.1.3). Tile plots using the package bipartite were also constructed to view which plant genera were being collected most often (Dormann et al., 2009). Data was not statistically compared between different groups, and instead we use this data to describe broad trends of bee foraging behavior.

### 2.2 Experiment group 2: Rearing *O. cornifrons* larvae on different diets to determine dietary tolerances

#### 2.2.1 Experiment 2A: Rearing *O. cornifrons* larvae on altered multifloral pollen diets

##### In vitro rearing and progeny outcomes

Modified rearing methods from Phan et al. (2020) were used to rear immature *O. cornifrons* in captivity. Five hundred mated adults sourced from the Penn State Fruit Research and Extension breeding program (Adams County, PA) were released at trap nests at a single site, the Penn State Arboretum (Site E, Centre County, PA; Table S1) in 4 weekly releases in May of 2020 (2000 adults total, 60:40 M:F). 200 eggs and larvae (up to 4th instar) were collected in May and June of 2020 from The Arboretum at Penn State (University Park, PA) for in vitro rearing. This methodology was changed in future experiments to only include eggs, rather than both eggs and larvae, in our study (see Data analysis for more details). There were 50 individuals per treatment group, but eggs or larvae suffering mechanical damage from transfer were removed from this pool. Paper inserts filled with pollen provisions, eggs, and larvae were collected every 48 h and replaced with new paper inserts to allow additional nesting. Eggs and larvae were transferred to prepared pollen provisions (220 μg, see Table 1) using a grafting tool (Dadant). Individual pollen provisions were placed in custom well plates with 8 mm diameter wells (Boyle & Pitts‐Singer, 2018). Plates were stored on open plastic trays and covered with foil to maintain humidity and temperature (~25°C, ~35% RH).

**TABLE 1:** Summary of experimental diets with P:L ratios used to assess larval diet suitability.

	Diet name	P:L ratio	Protein μg/mg	Lipid μg/mg	Sample size
Altered multifloral diets (2020)	Control (unaltered pollen)	5.5:1	191.40	34.40	44
	Modified control	5.5:1^a^	327.72	59.54	42
	High lipid diet	0.4:1	191.40	443.18	42
	Mid‐range diet	6.6:1	228.30	34.40	48
	High protein diet	14.5:1	498.36	34.40	33
Monofloral diets (2022)	Eastern black walnut (*Juglans nigra*)	0.6:1	51.38	81.63	35
	Ribwort plantain (*Plantago lanceolata*)	1.4:1	125.23	87.64	35
	Black willow (*Salix nigra*)	2.5:1	115.90	46.22	35
	Orchard grass (*Dactylis glomerata*)	4.2:1	111.09	26.33	35
	Red maple (*Acer rubrum*)	6.3:1	217.76	34.38	35
	Control (mixed *O. cornifrons* pollen provisions)	3:1^b^	–	–	35
Naturally different multifloral diets (2022)	High lipid diet	1:1	83.17	82.73	35
	Mid‐range diet 1	2.9:1	87.95	29.61	35
	Mid‐range diet 2	5.2:1	77.51	14.90	35
	High protein diet	10.5:1	104.62	9.88	35
	Control (homogenized *O. cornifrons* pollen provisions)	3:1^b^	–	–	35

*Note*: Eggs or immature larvae were transferred from their original pollen diet to experimental diets while survival and development time was monitored.

^a^ Modified by adding protein and lipids to change the nutrient concentrations without impacting the overall P:L ratio.

^b^ Diets were presumed to be a 3:1 ratio after homogenization as this was the mean P:L ratio from previous analyses of *O. cornifrons* pollen balls.

Pollen diets were created by mixing a base multifloral pollen collected by honey bees (CC Pollen Co.) with protein (casein powder; Sigma Aldritch) and/or lipids (canola oil), as in Vaudo et al. (2016). Diets were also moistened with 20% sucrose solution until pollen reached a dough‐like consistency to provide bees with sufficient carbohydrates and moisture. However, honey bee corbicular pollen is already mixed with nectar, and thus the carbohydrate concentration may not be consistent across experiments (Thorp, 1979). Pollen was homogenized in a standard coffee grinder prior to mixing. The base pollen diet used had a starting P:L ratio of 5.5:1. Diets consisted of an unaltered control pollen (5.5:1), a low P:L ratio pollen (0.4:1), a mid‐range P:L ratio pollen (6.6:1), a high P:L ratio pollen (14.5:1), or a modified control diet (5.5:1). This modified control was the same base pollen used for other diets with protein (casein powder) and lipids (canola oil) added to maintain a 5.5:1 ratio but increase the overall concentrations of these two macronutrients: this allows us to test the tolerance of bees to these additives. Diet recipes with the amount of added lipids and proteins can be found in Table S2. Example pollen alteration calculations can be found in Crone and Grozinger (2021). Bees were placed in cold storage in a standard lab refrigerator (VWR international, Radnor, PA), at 4°C following pupation and remained until the following spring (140 days). Bee progeny outcomes, in response to each diet, were measured by recording larval mortality (if bees completed spinning their cocoons) and adult mortality (if bees were alive the following spring, confirmed by manual extraction from cocoons).

##### Data analysis

We aimed to only use eggs in our in vitro rearing experiments to prevent skewed results from older larvae, but 2020 was a poor year for *O. cornifrons* production due to weather patterns (high variation in temperatures during the nesting period). Thus, for this particular experiment, both eggs and larvae were used (the other experiments used eggs only). Variation in larval development was accounted for by taking the mass of each larva prior to transferring them to randomized diet treatments. In the final analysis we included all larvae below 25 mg (*n* = 209 total, exact sample counts in Table 1) to remove larger larvae and allow for even sample sizes across treatment groups. A generalized linear model with a binomial distribution and logarithmic linkage was used to determine if there were significant differences in survival across treatment groups, with diet treatment group as the predictor variable and survival as the outcome variable. Starting mass and day of graft (DOG) were also included as predictor variables to determine if they also significantly impacted survival. Starting mass (*p =* .00032, estimate = −0.12, SE = 0.03, *χ* ^2^ = 89.13, df = 6) and DOG (*p* = .01762, estimate = 0.59, SE = 0.25, *χ* ^2^ = 89.13, df = 6) significantly impacted results. In this binomial model, 1 = death and 0 = survival, and results indicate that larger larvae were more likely to survive diet transfer, and thus, should be used when interpreting these results. This information was used to design experiment 1B, where eggs that hatched on the same day were evenly distributed across dietary treatment groups rather than eggs and larvae.

#### 2.2.2 Experiment 2B: Rearing *O. cornifrons* larvae on monofloral and multifloral pollen diets

##### In vitro rearing and progeny outcomes

In 2022, 350 *O. cornifrons* eggs were collected from a single site, the Rock Springs Experimental Farm (Site G, Table S1). One thousand four hundred mated adults (M:F 2:1) sourced from this location in the prior year were released periodically prior to collection. Paper inserts filled with pollen provisions and eggs were collected every 48 h and replaced with new paper inserts to allow additional nesting. Eggs were transferred as they became available (35 per treatment group total) to monofloral and multifloral diets mixed with 1:1 Pro‐Sweet:water solution (Mann Lake) in custom well plates, as described in experiment 1A. Pro‐Sweet is a sugar substitute commonly used in beekeeping that does not ferment and includes fructose (22%), dextrose (27%), sucrose (50%), maltose (0.5%), and higher saccharides (0.5%), which is more similar to nectar composition than sucrose alone (Chalcoff et al., 2006). Individuals were housed in a walk‐in environmental chamber (~24°C, 60–70 RH%). Experimental diets were composed of (1) pollen from individual plant species (Stallergenes Greer, Lenoir, NC), (2) naturally different multifloral pollen mixes collected by honey bees (collected from colonies in Penn State apiaries in Centre County, PA), or (3) a control diet composed of 30 homogenized and randomly selected pollen balls collected from *O. cornifrons* nests (Table 1). Single plant species diets came with a laboratory purity report, and included *Juglans nigra*, *Plantago lanceolata*, *Salix nigra*, *Dactylis glomerata*, and *Acer rubrum*. Control provision masses were collected from completed *O. cornifrons* nests on the start date of each new experimental diet transfer. The control group was presumed to have a P:L ratio of 3:1, as this was the mean P:L ratio found across sites and weeks in Experiments 1A in 2019, but this was not formally evaluated. Progeny outcomes of individuals were measured via larval survival (bees that completed cocoons), larval developmental timing (time between egg hatching and cocoon completion), pupation survival, and time to pupation (time between cocoon completion and pupation; Barraud et al., 2022; Eckhardt et al., 2014). Bees were observed each day for developmental changes. X‐radiography (Multifocus Digital Radiography System; Faxitron Bioptics) was used to view bee pupation after cocoon completion (4.5 s exposure at 25 kVp).

There was background mortality (*n* = 35/350 or 10% total mortality across treatment groups) throughout the experiment due to an unknown fungal pathogen. Signs and symptoms were consistent with chalkbrood (Mader et al., 2010), but pathogen identity was not confirmed via PCR. As chalkbrood is easy to identify visually, once these bees were removed from the original sample pool it is unlikely that chalkbrood further impacted survival (Jensen et al., 2013). Nineteen uninfected samples were randomly chosen from the larger dataset for each diet treatment for statistical analyses.

##### Data analysis

A generalized linear model with a binomial distribution and logarithmic linkage was used to determine if there were significant differences in survival across treatment groups. Data from bees fed naturally different multifloral and monofloral diets were fit in separate models with the control diet. Diet was a fixed predictor variable and survival was the outcome variable. DOG was also included as a predictor variable and was not found to be a significant predictor of survival for bees fed multifloral (estimate = 0.05, *p* = .1045, SE = 0.035, *χ* ^2^ =0.37, df = 5) or single source diets (estimate = 0.46, *p* = .0559, SE = 0.2408, *χ* ^2^ = 74.31, df = 6). A Kruskal–Wallis rank sum test was then used to determine if there were significant differences in time to cocoon completion or pupation between treatment groups.

## 3 RESULTS

### 3.1 Experiment 1A: Examining nutritional content of pollen provisions across sites and time

**FIGURE 1:**
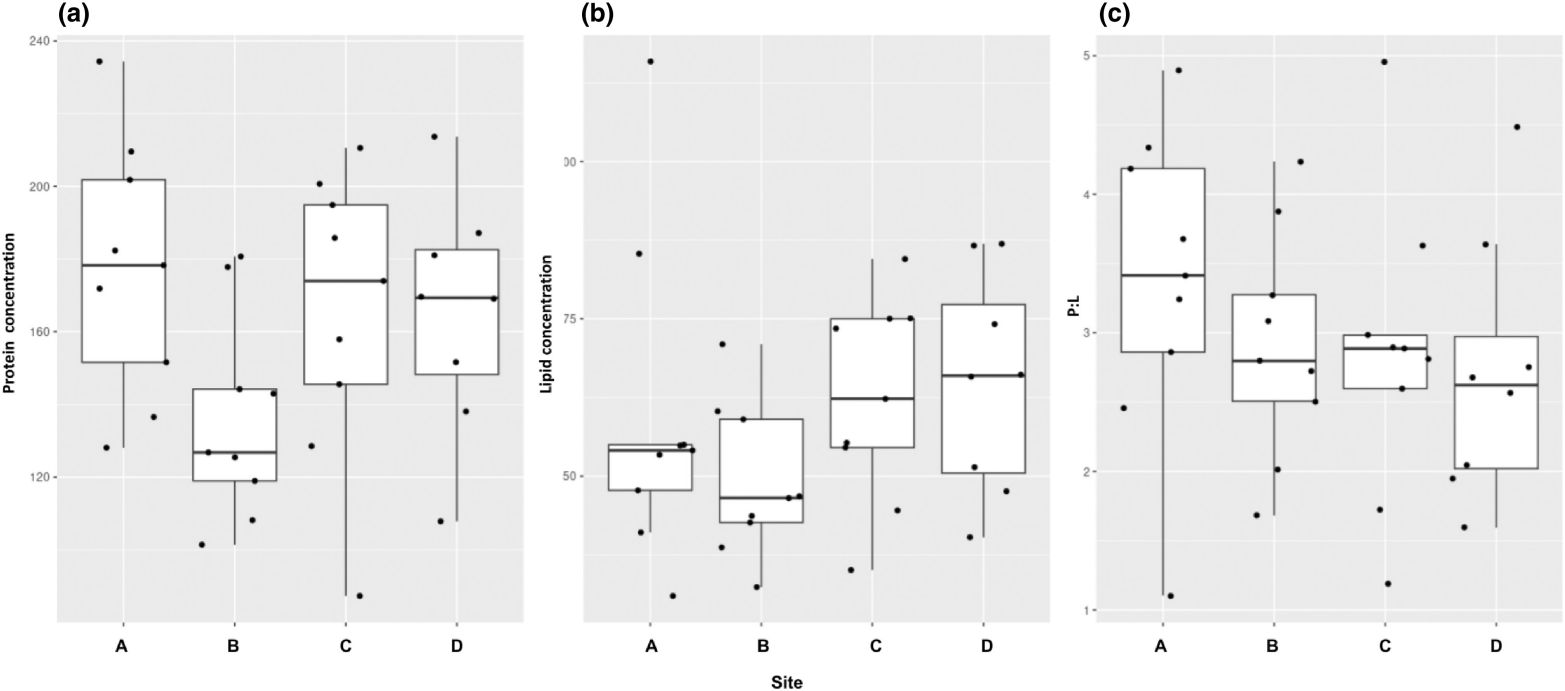
Nutritional content of *Osmia*‐collected larval provisions across locations. Whole pollen provision protein concentrations (a), lipid concentrations (b), and P:L ratios (c) across sites sampled in September of 2019 from the Penn State Fruit Research and Extension Center. Sites A, C, and D were orchard sites while site B was a suburban site. Pollen balls were unconsumed and represented provisions generated across an entire season. Only one pollen ball was analyzed per nest or individual.

There were no significant differences in protein concentration, lipid concentration, or P:L ratio across sites (Figure 1, see Table S3 for all pairwise comparisons and sample sizes). Overall, the protein concentration ranged from 87.37 to 234.37 μg/mg (mean 160.69 ± 6.07 μg/mg), the lipid concentration ranged from 30.96 to 115.92 μg/mg (mean 58.80 ± 3.16 μg/mg), and P:L ratio ranged from 1.10 to 4.89:1 (mean 2.96:1 ± 0.16) across all samples.

**FIGURE 2:**
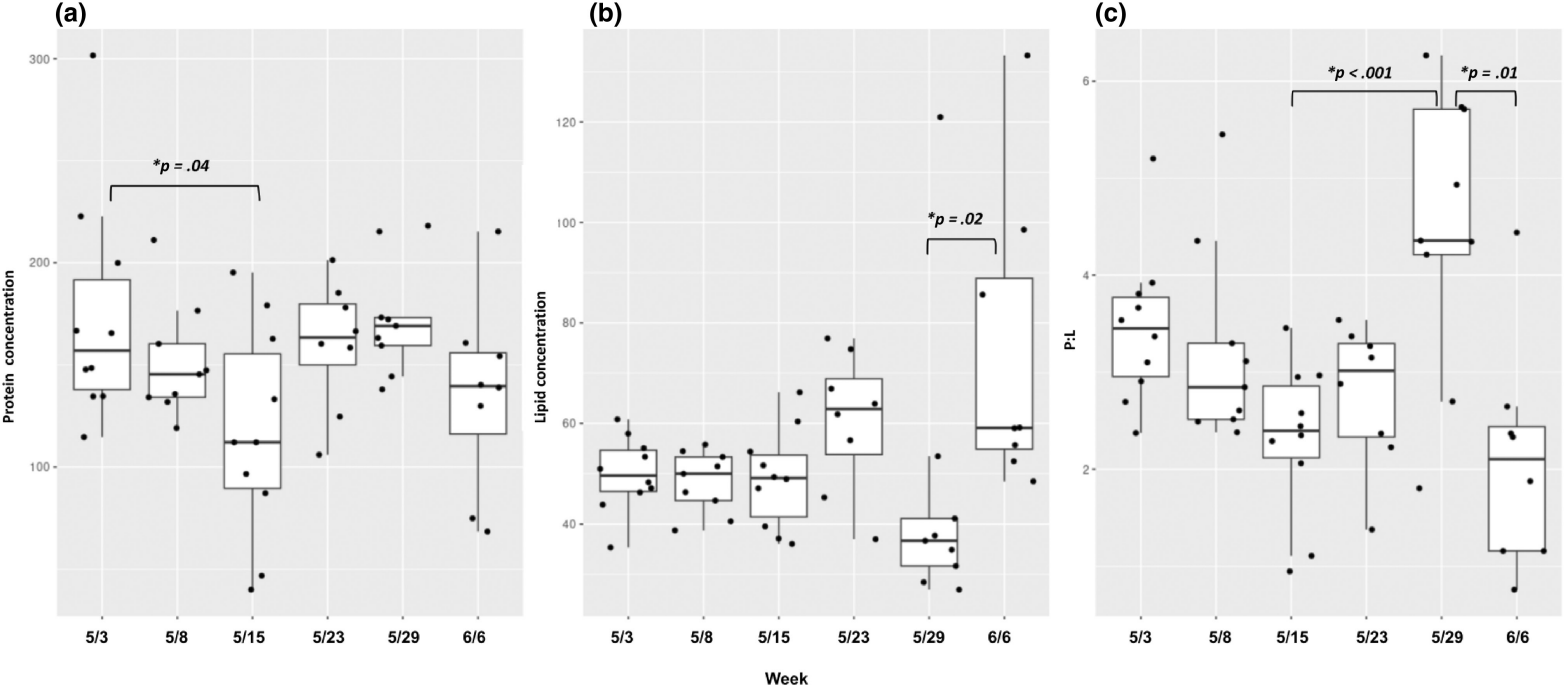
Nutritional content of Osmia‐collected larval provisions across time. Whole pollen provision protein concentrations (a), lipid concentrations (b), and P:L ratios (c) across 6 weeks (May 3rd–June 6th, 2019). Pollen was collected weekly from the Arboretum at Penn State.

While there were no significant differences in the nutritional content of pollen provisions across sites, there were differences between timepoints in samples collected at a single site (Figure 2, see Table S3 for all pairwise comparisons). Protein concentration, lipid concentration, and P:L ratio all varied significantly (Figure 2). Overall, the protein concentration spanned the range from 39.95 to 301.61 μg/mg (mean 151.42 ± 6.22 μg/mg), the lipid concentration spanned the range from 26.97 to 133.26 μg/mg (mean 53.94 ± 2.69 μg/mg), and P:L ratio spanned the range from 0.75 to 6.26:1 (mean 3.06:1 ± 0.17) across all samples. Pollen provisions collected during week 3 had lower protein concentrations than those collected during week 1, while pollen provisions collected during week 5 had lower lipid concentrations than those from week 6. Provision P:L ratios from pollen collected in week 5 were higher than those from weeks 3, 4, and 6.

### 3.2 Experiment 1B: Examining the genera richness and composition of whole pollen provisions and collections from individual foraging trips

**FIGURE 3:**
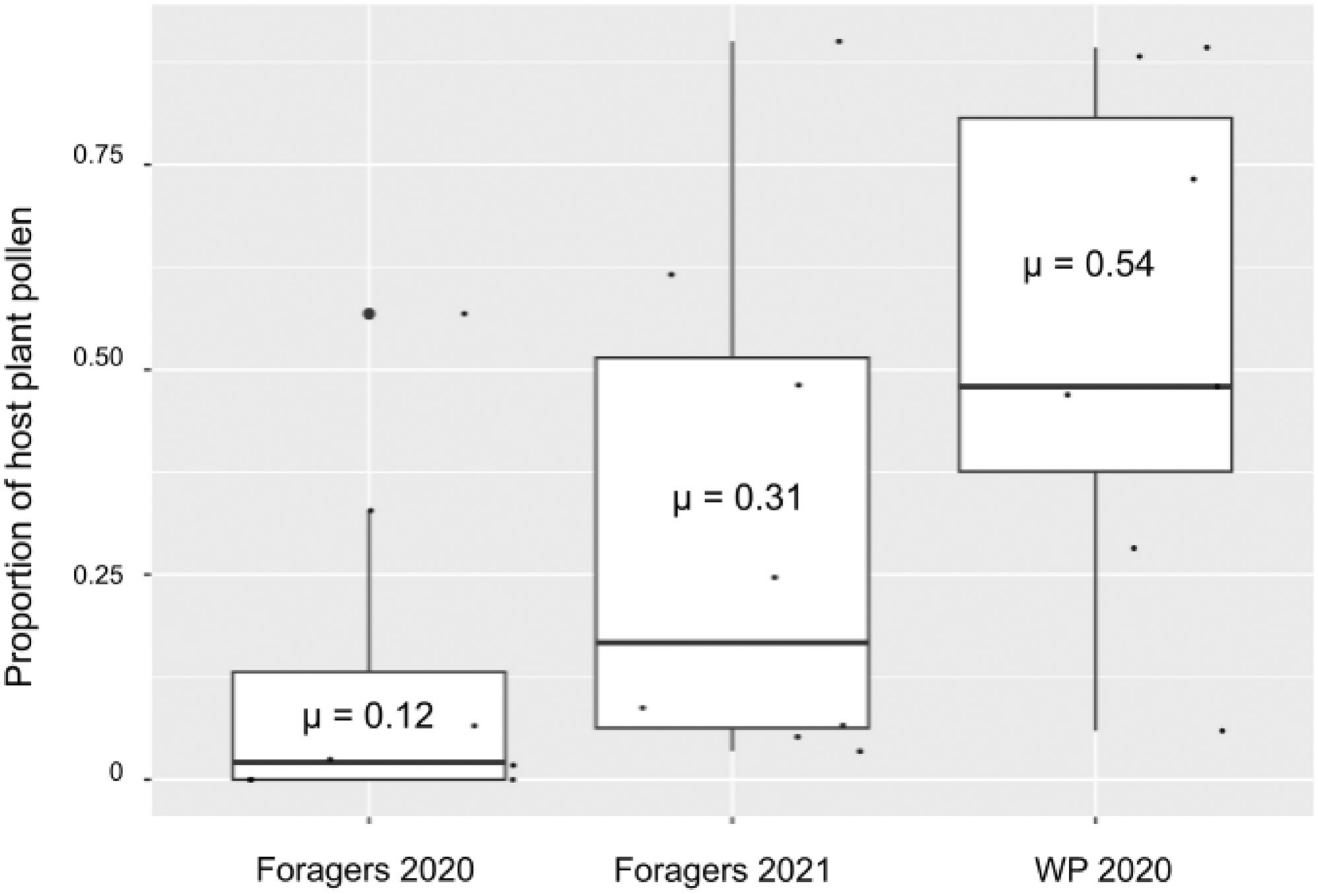
Proportion of host plant pollen in samples collected from individual foraging trips and whole pollen provisions (WP). Whole provisions, which represent many pollen foraging trips, had a mean proportion of 0.54 host plant pollen. This indicates that nearly half of whole provisions are composed of non‐host plant pollen.

Whole pollen provisions and individual foraging trip samples consisted of plants from many different plant genera, with a mean of 3.62 ± 1.51 genera (range = 3–5, *n* = 8) from individual forager trips in 2020, a mean of 8.37 ± 0.74 genera (range = 5–14, *n* = 8) from individual forager trips in 2021, and a mean of 3.42 ± 3.46 genera (range = 2–6, *n* = 7) from whole pollen provisions in 2020 (Figure 3).

**FIGURE 4:**
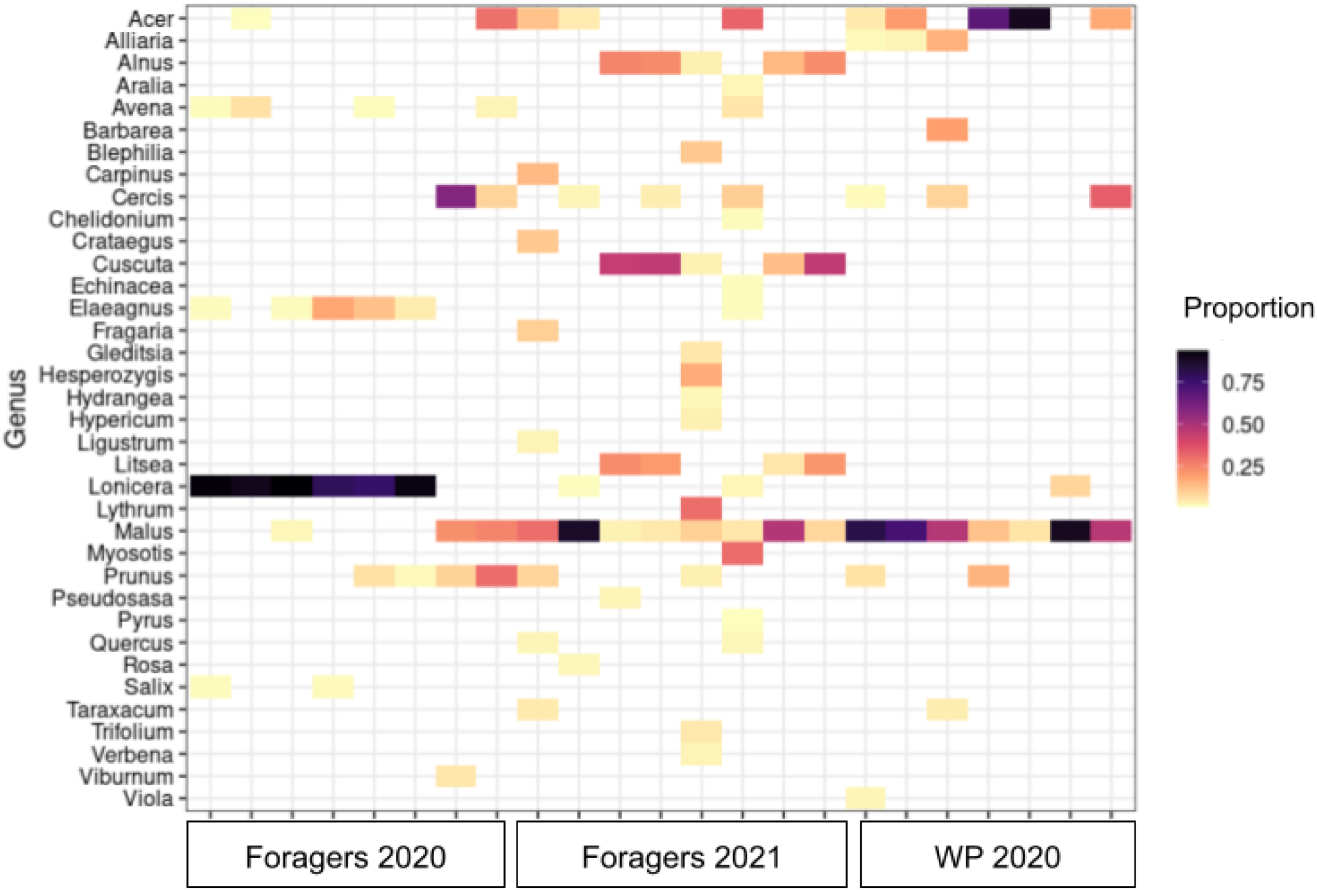
Plant genera composition of individual foraging trips and whole pollen provisions (WP). Individual samples are represented as columns. Tiles shaded purple have higher relative proportions than pink and yellow tiles (see Supporting Information for exact proportions). Genera included made up >1% of a single pollen sample.

The proportion of different plant genera present in pollen collections from foragers in 2020, foragers in 2021, and whole pollen provisions in 2020 are also presented here (Figure 4). Genera *Clusia*, *Hirtella*, and *Parinari* were excluded from these analyses as these plants are not present in Pennsylvania and were thought to be mismatches in the database. Genera that made up >1% of plant sequenced reads in each sample are included, and each column represents a single pollen sample (*n* = 8 for foragers in 2020 and 2021, respectively, and *n* = 7 for whole provisions in 2020). Statistical tests were not conducted for this data, but broadly, the plants collected most were *Acer*, *Cercis*, *Lonicera*, and *Malus*.

The proportion of pollen from host plant genera from individual forager trips and whole pollen provisions were also assessed. Foragers in 2020 had a mean host plant pollen proportion of 0.12 ± 0.21, while foragers in 2021 had a mean host plant pollen proportion of 0.31 ± 0.32. Whole pollen provisions had a mean proportion of 0.54 ± 0.31 pollen from host plants.

### 3.3 Experiment 2A: Rearing *O. cornifrons* larvae on altered multifloral pollen diets

**FIGURE 5:**
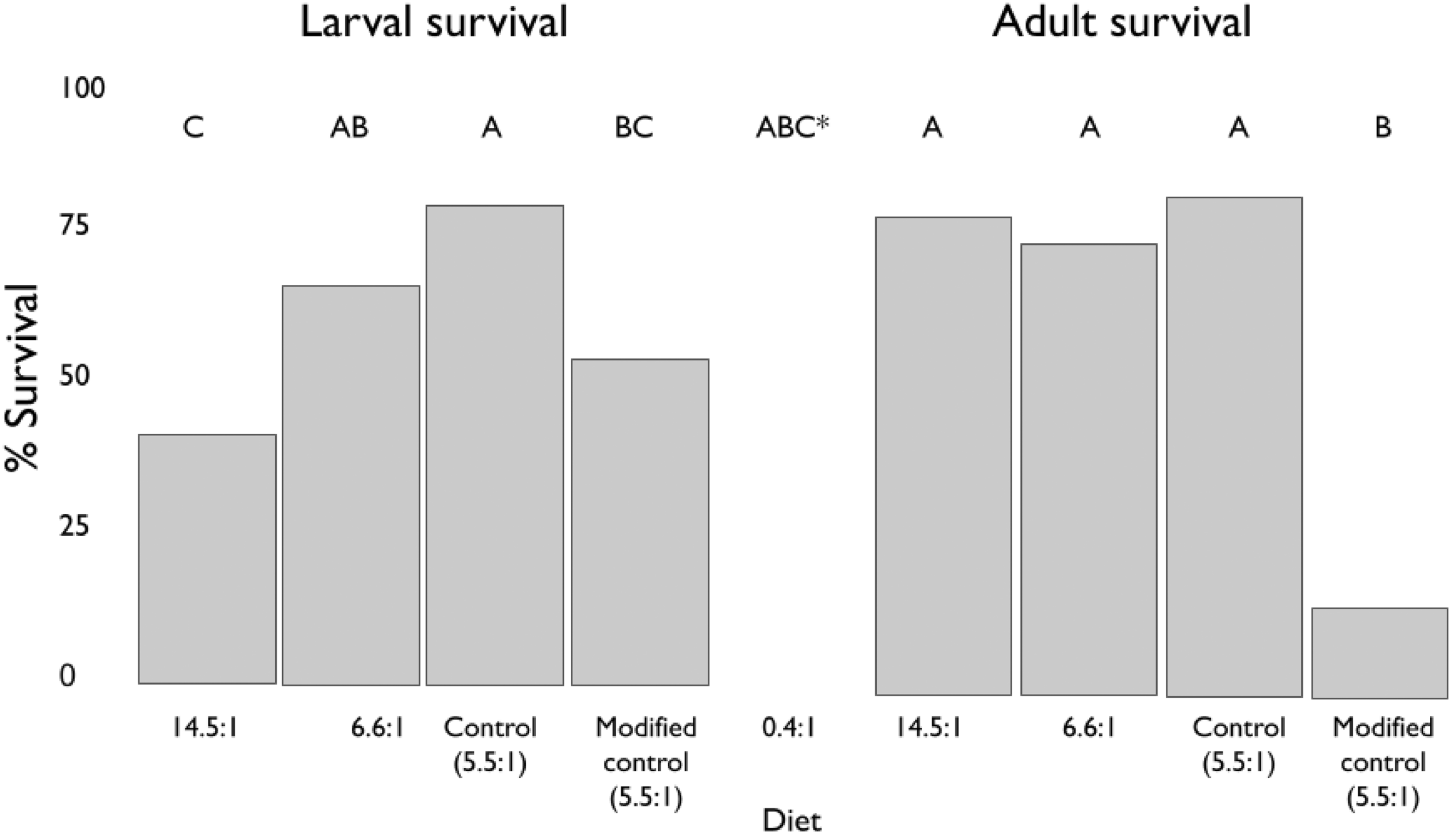
The percent survival of larvae and adults in each dietary treatment group. Treatment groups on the *X*‐axis are dietary P:L ratios, and percent survival (percent cocoons spun for larvae and percent pupated for adults) is on the *Y*‐axis. Larval and adult models were fit separately. The adult model only includes bees that survived the larval stage. *Larvae fed the low P:L ratio diet (0.4:1) are not truly significantly different from other treatment groups due to zero surviving individuals to compare with other groups in the GLM (*p* = 1).

Larvae fed the high lipid diet (0.4:1) had the lowest survival rate of all treatment groups with 0 surviving individuals (Figure 5). Larvae fed the control (5.5:1) and mid‐range diets (6.6:1) had higher survival than those fed the high protein diet (14.5:1). Larvae fed the control (5.5:1) diet also had a higher survival rate than the modified control (5.5:1). Bees that survived until the following spring were considered successful in the model of adult survival (Figure 5). DOY and starting mass were also included in the adult model as predictor variables. Bees fed the high protein diet (14.5:1), the mid‐range diet, and the control diet (5.5:1) all had higher survival rates than those fed the modified control diet (5.5:1; see Table S4 for pairwise comparisons). There were 200 total individuals and 50 per treatment group.

### 3.4 Experiment 2B: Rearing *O. cornifrons* larvae on monofloral and naturally different multifloral pollen diets

**FIGURE 6:**
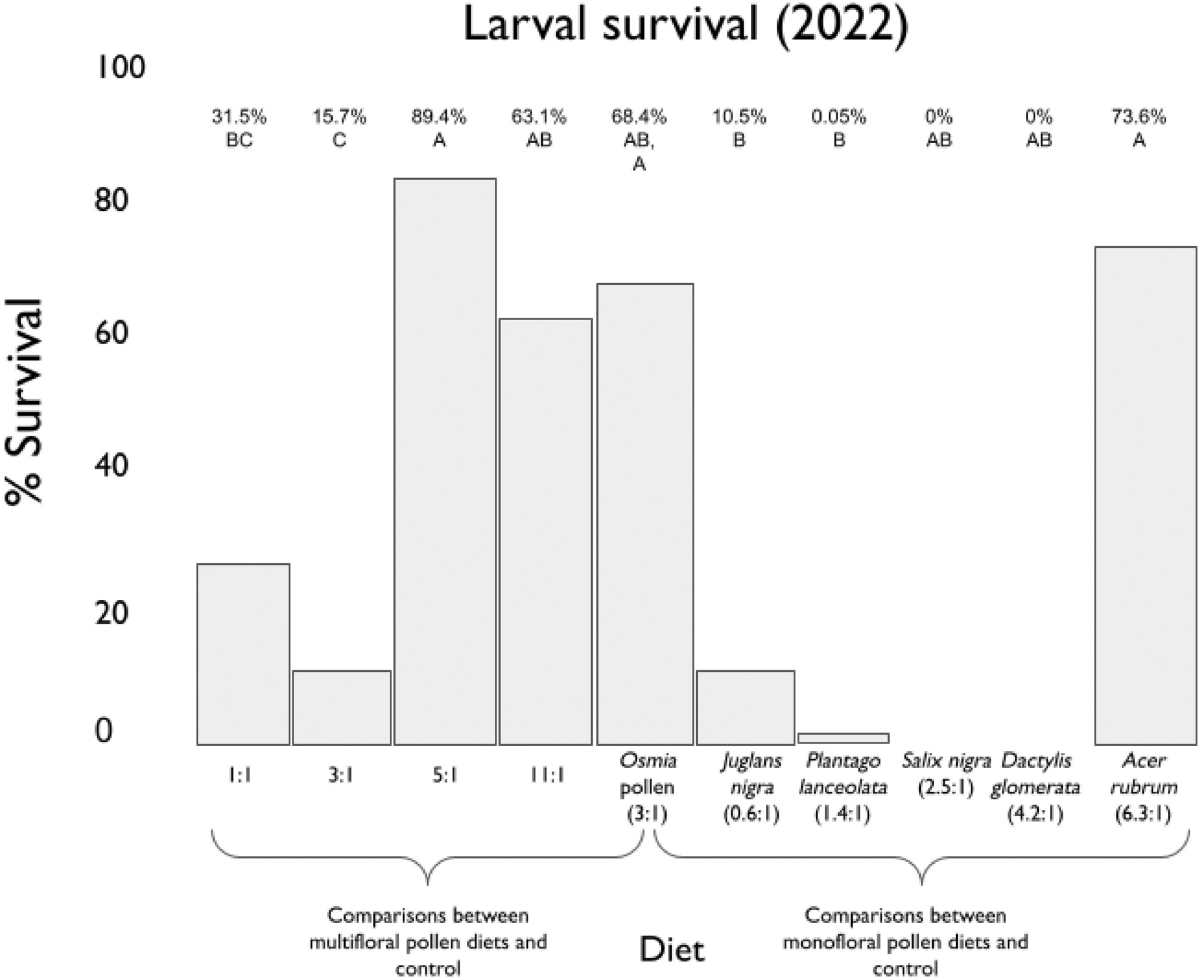
The percent survival of larvae in each dietary treatment group. Treatment groups on the *X*‐axis are dietary P:L ratios, and percent survival (percent cocoons spun by larvae) is on the *Y*‐axis. Models were fit separately for multifloral pollen diets and monofloral pollen diets.

When comparing the multifloral pollen diets, larvae fed the control diet (3:1), the high protein (10.5:1) diet, and mid‐range 1 (5.2:1) diet had higher rates of survival than those fed the mid‐range 2 (2.9:1) diet and did not differ from one another (Figure 6). Larvae fed the mid‐range 1 (5.2:1) diet also had higher survival rates than those fed the high lipid (1:1) diet. Bees fed the high lipid (1:1) and mid‐range 2 (2.9:1) diets did not have different survival rates (see Table S5 for pairwise comparisons).

When comparing the monofloral pollen diets, larvae in the control group (3:1) had a higher rate of survival than those in the groups fed *Juglans nigra* (0.6:1) and *Plantago lanceolata* (1.4:1). *Acer rubrum* (6.3:1) was the only monofloral diet that led bees to have the same level of survival as the control diet (3:1). None of the bees fed *Salix nigra* (2.5:1) or *Dactylis glomerata* (4.2:1) survived. In addition, there was no correlation between protein concentration (*R* = .14), lipid concentration (*R* = .25), or P:L ratio (*R* = .25) and survival. There were no significant differences in time to cocoon spinning among treatment groups (Figure S1).

**FIGURE 7:**
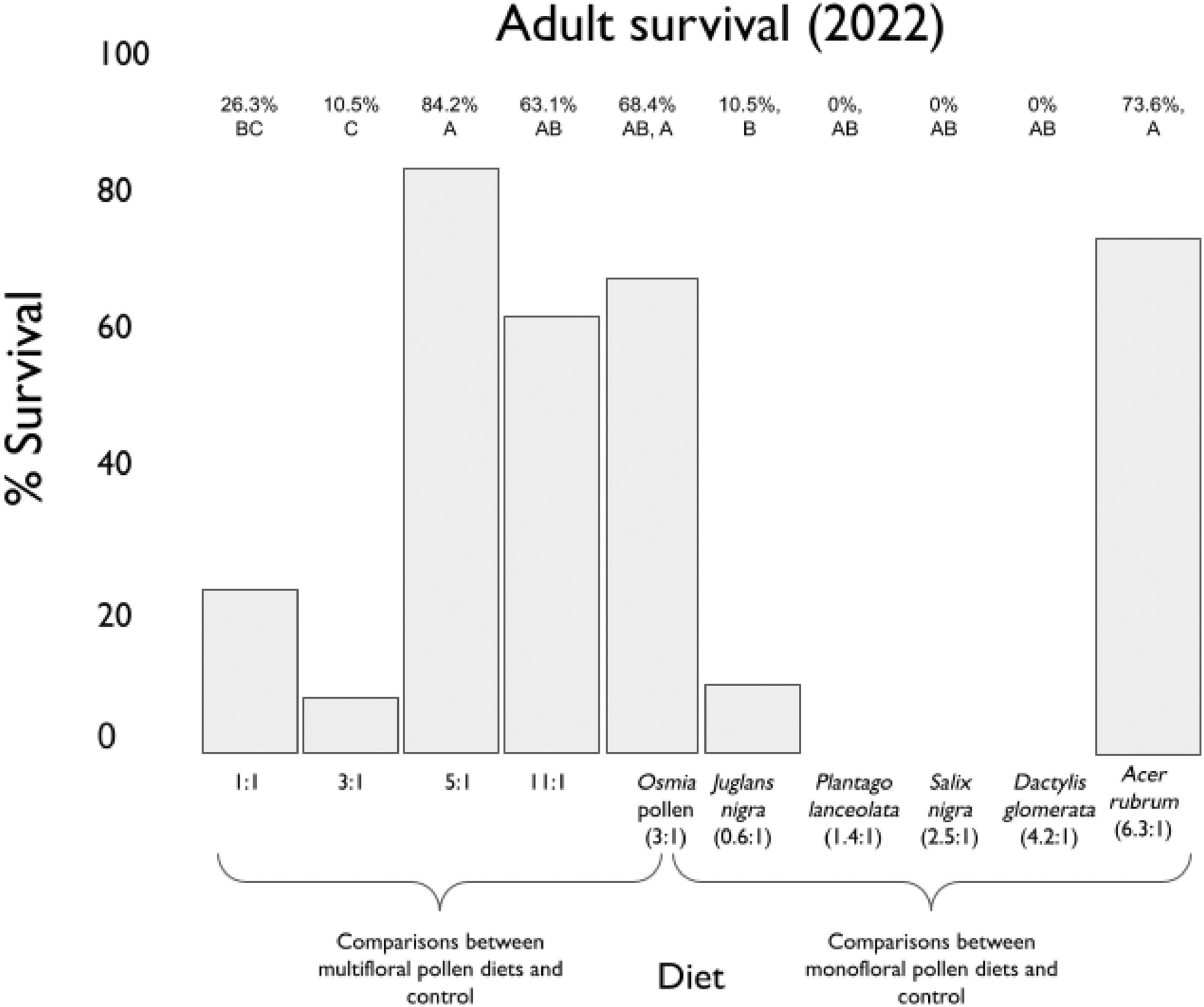
The percent survival of adults in each dietary treatment group. Treatment groups on the *X*‐axis are dietary P:L ratios, and percent survival (percent of pupated adults) is on the *Y*‐axis. Models were fit separately for multifloral pollen diets and monofloral pollen diets.

For bees in the multifloral diet groups, larvae that consumed the control diet (3:1) did not have different rates of survival than those that consumed the high protein (10.5:1) or mid‐range 1 (5.2:1) diets (Figure 7). Larvae fed these diets had higher rates of survival than those fed the mid‐range 2 (2.9:1) diet. Larvae that consumed the high lipid (1:1) diet had the same survival rate as larvae in the mid‐range 2 (2.9:1), high protein (10.5:1), and control (3:1) diet groups, but did have a lower rate of survival than larvae in the mid‐range (5.2:1) diet group. For bees in the monofloral diet groups, there were no surviving pupae in groups fed *Plantago lanceolata* (1.4:1), *Salix nigra* (2.5:1), or *Dactylis glomerata* (4.2:1). Larvae fed *Acer rubrum* (6.3:1) had a higher rate of pupation than larvae fed *Juglans nigra* (0.6:1). There were no significant differences in time to pupation among treatment groups (Figure S2).

## 4 DISCUSSION

Our pollen nutritional surveys show that dietary protein concentrations, lipid concentrations, and P:L ratio content in *O. cornifrons* pollen collections are surprisingly broad, with an overall average P:L ratio of 3.02:1 and a range of 0.75–6.26:1 when datasets across different sites and time periods were combined. These results demonstrate that *O. cornifrons*' mean target P:L ratio is relatively consistent across studies (in comparison with Vaudo, Tooker, et al., 2020), as hypothesized, but that diet breadth is broader than predicted. When comparing collections between time periods, there were some differences in pollen protein concentration, lipid concentration, and P:L ratio. This may be due to changing plant communities (in species richness and identity) during the shift from early spring to summer, but as we did not collect data on what plants were in bloom during each time period, additional studies with consistent plant surveys and timed pollen provision collections are required to test this hypothesis. It is also possible that P:L ratio requirements can change intra‐specifically over time, as insects' nutritional needs can change in times of stress (Cotter et al., 2011; Crone & Grozinger, 2021) or during different stages of life (DeGrandi‐Hoffman et al., 2018). There were no differences in protein concentration, lipid concentration, or P:L ratio between sites, even though one site was in a suburban area while the others were in orchard or wooded areas.

The in vitro rearing experiments conducted to further explore what could be considered the ideal target dietary P:L ratio (i.e. lowest mortality, fastest developmental rate) and tolerable niche breadth encompassing this target demonstrated that *O. cornifrons* larvae could tolerate a wide breadth of P:L ratios, but factors other than P:L ratio clearly played an important role in developmental outcomes. We found that the protein and lipid additives used previously to alter honey bee (*A. mellifera*; Crone & Grozinger, 2021) and bumble bee (*B. impatiens*; Vaudo et al., 2016) diets for nutritional experiments were not suitable for *O. cornifrons* health and development: addition of canola oil to increase lipid content seemed to be especially problematic. In addition, these experiments included larvae, and the original diet these bees were transferred from could have rescued individuals on a poorer diet from mortality. Therefore, here we discuss only the experimental trials that tested multifloral and single source diets. For the multifloral diets (honey bee collected pollen), *O. cornifrons* were able to develop successfully on diets ranging from 3:1 to 10.5:1 (presuming the control diet has a 3:1 ratio). However, single source pollen from *Dactylis glomerata* (4.2:1) fell within this range, and this diet was not able to support successful *O. cornifrons* development. Moreover, other multifloral and single source diets that fell within the range of field‐collected provisions (1:1 and 2.9:1 for *Plantago lanceolata* and *Salix nigra*, respectively) also did not support development. Previous work has found that *O. cornuta* must balance pollen collections across plant species to control for levels of toxic secondary plant compounds (Eckhardt et al., 2014), and compounds such as these could have impacted survival in our experiments. In addition, other studies have found that micronutrients must also be balanced appropriately to create a quality diet for *Osmia* spp., and that pollen from a diversity of plants does not always provide vital nutrient availability (Filipiak et al., 2022; Filipiak & Filipiak, 2020). Finally, the pollen microbiome also makes up a significant portion of the nutrients bees obtain from pollen (Steffan et al., 2019). While the multifloral diets collected by honey bees would have a diverse microbiome (Prado et al., 2022), the single source diets obtained from a commercial lab are likely much more limited. This suggests that while pollen P:L ratio niche breadth is broad, pollen with a P:L ratio that falls within this window alone is not enough to create a quality diet for *O. cornifrons*, and the plant species that make up these diets must also be carefully considered.

Caution should also be used when interpreting these results, particularly for lipid tolerance and preferences. Prior studies indicate that Omega‐6:3 ratios are more important than overall lipid levels for honey bee memory and learning (Arien et al., 2018). Furthermore, bumble bees perceive fatty acid concentrations, but not amino acid concentrations, when making foraging decisions (Ruedenauer et al., 2020). Therefore, it may be possible that diet suitability in our study was partially driven by these important individual compounds, and certain nuances are unable to be examined with our data. Future studies should assess these finer‐scale differences in more detail.

Pollen from individual foraging trip collections showed pollen from many different plant genera, demonstrating some form of foraging decision making or risk–reward evaluation. This could indicate that *O. cornifrons* are balancing pollen nutrients (e.g. macronutrients, micronutrients, toxic secondary plant compounds) from different plant genera within foraging trips, although floral availability and resource distribution also likely influence this behavior, and further study would be required to support this hypothesis. It is important to note that plant visitation patterns may also be due to other plant cues that bees have co‐evolved with, such as floral scent, color, or shape, and may not be related to pollen nutritional content (Baracchi, 2019). Foragers collected in 2021 (later in the growing season) also appeared to have a distribution that ranged higher in the number of plant genera pollen was collected from than individual foraging trip collections in 2020, suggesting that *O. cornifrons* may shift their preferences throughout the season and/or their lifetime to obtain a greater diversity of plants as they become available, or niche partition with other bee species. An alternate hypothesis is that bees simply move on to the next best plant once their main host plants cease blooming, which could explain why Lonicera was the most important resource in 2020, while Malus was most important in 2021. As the present study was unable to formally evaluate these hypotheses, additional work comparing *O. cornifrons* collections to other bee species in the same pollinator community would be needed to explore these hypotheses.

Whole pollen provisions had a wide range in proportions of host plant pollen, with some provisions composed of as low as 5% host plant pollen, and others within the range above 70% (similar to Vaudoet al., 2020 with a mean of 70.25%). While it is possible that bees with low host pollen proportions did not have access to enough host plants in the environment, this seems unlikely given that all whole pollen provisions were collected from the same location and date. This may indicate that there is a larger degree in dietary variability than previously observed for *O. cornifrons*. Indeed, Klečka et al. (2022) and Gaiarsa et al. (2022) demonstrated that generalist bee species in genus Ceratina are composed of many specialist individuals, and with more advanced DNA metabarcoding technology to track plant‐pollinator interactions, future studies can glean more insight into individual variation within a species. Furthermore, this brings into question if *O. cornifrons* can truly be categorized as mesolectic. Non‐host plant genera that bees primarily collected pollen from were Lonicera and Acer. Indeed, bees performed well when reared in vitro on Acer rubrum pollen alone. While we did not measure the nutritional properties of pollen from these plants in the present study, it is possible that the plants from these genera are already “balanced” in terms of micronutrients or secondary plant compounds (Filipiak et al., 2022; Filipiak & Filipiak, 2020), and that other plants that make up the rest of the provision are carefully proportioned to meet nutritional needs and prevent toxicity.

## AUTHOR CONTRIBUTIONS

**Makaylee K. Crone:** Conceptualization (equal); formal analysis (lead); funding acquisition (supporting); investigation (lead); methodology (equal); visualization (lead); writing – original draft (lead); writing – review and editing (equal). **Natalie K. Boyle:** Conceptualization (equal); methodology (equal); project administration (supporting); supervision (equal); writing – original draft (supporting); writing – review and editing (equal). **Sean T. Bresnahan:** Software (lead); validation (lead); writing – original draft (supporting); writing – review and editing (equal). **David J. Biddinger:** Conceptualization (supporting); methodology (supporting); project administration (supporting); supervision (supporting); writing – review and editing (equal). **Rodney T. Richardson:** Software (equal); validation (supporting); writing – original draft (supporting); writing – review and editing (supporting). **Christina M. Grozinger:** Conceptualization (equal); funding acquisition (equal); methodology (equal); project administration (equal); resources (lead); supervision (lead); writing – review and editing (equal).

## FUNDING INFORMATION

This work was supported by funding from Wyman's of Maine to the Penn State Center for Pollinator Research, the Penn State Bunton Waller Program, and the National Science Foundation Graduate Research Fellowship Program [DGE1255832]. Any opinions, findings, and conclusions or recommendations expressed in this material are those of the authors and do not necessarily reflect the views of the National Science Foundation.

## CONFLICT OF INTEREST STATEMENT

The authors have no competing interests to declare.

## Supporting information


Data S1

## Data Availability

All numerical data are available in the Dryad repository at https://datadryad.org/stash/share/b6OCJe14F6sdlL3I_eaMN‐1FdF‐qbWbTHzkL5aLnSlA (Reviewer link). Sequencing outputs have been archived on NCBI Sequence Read Archive under Bioproject SUB13689831. Custom code for bioinformatics analyses can be found at https://github.com/RTRichar/SimpleSequenceClassification. MetaCleaner code and guidelines can be found at https://github.com/sbresnahan/metacleaner.

## References

[ece310640-bib-0001] Arien, Y. , Dag, A. , & Shafir, S. (2018). Omega‐6:3 ratio more than absolute lipid level in diet affects associative learning in honey bees. Frontiers in Psychology, 9, 1001. 29971031 10.3389/fpsyg.2018.01001 PMC6018467

[ece310640-bib-0002] Artz, D. R. , Allan, M. J. , Wardell, G. I. , & Pitts‐Singer, T. L. (2014). Influence of nest box color and release sites on *Osmia lignaria* (Hymenoptera: Megachilidae) reproductive success in a commercial almond orchard. Journal of Economic Entomology, 107, 2045–2054. 26470068 10.1603/EC14237

[ece310640-bib-0003] Baracchi, D. (2019). Cognitive ecology of pollinators and the main determinants of foraging plasticity. Current Zoology, 65, 421–424. 31423133 10.1093/cz/zoz036 PMC6688568

[ece310640-bib-0004] Barraud, A. , Barascou, L. , Lefebvre, V. , Sene, D. , Le Conte, Y. , Alaux, C. , Grillenzoni, F.‐V. , Corvucci, F. , Serra, G. , Costa, C. , Vanderplanck, M. , & Michez, D. (2022). Variations in nutritional requirements across bee species. Frontiers in Sustainable Food Systems, 6, 824750.

[ece310640-bib-0005] Behmer, S. T. , & Joern, A. (2008). Coexisting generalist herbivores occupy unique nutritional feeding niches. Proceedings of the National Academy of Sciences of the United States of America, 105, 1977–1982. 18238894 10.1073/pnas.0711870105 PMC2538867

[ece310640-bib-0006] Bloom, E. H. , Oeller, E. C. , Olsson, R. L. , Brousil, M. R. , Schaeffer, R. N. , Basu, S. , Fu, Z. , & Crowder, D. W. (2022). Documenting pollinators, floral hosts, and plant–pollinator interactions in U.S. Pacific Northwest agroecosystems. Ecology, 103, e3606. 34897664 10.1002/ecy.3606

[ece310640-bib-0007] Boyle, N. , & Pitts‐Singer, T. (2018). In vitro larval rearing of Osmia lignaria (Hymenoptera: Megachilidae) for diet manipulation studies. Entomological Society of America.

[ece310640-bib-0008] Burkle, L. A. , Marlin, J. C. , & Knight, T. M. (2013). Plant‐pollinator interactions over 120 years: Loss of species, co‐occurrence, and function. Science, 339, 1611–1615. 23449999 10.1126/science.1232728

[ece310640-bib-0009] Chalcoff, V. R. , Aizen, M. A. , & Galetto, L. (2006). Nectar concentration and composition of 26 species from the temperate forest of South America. Annals of Botany, 97, 413–421. 16373370 10.1093/aob/mcj043 PMC2803636

[ece310640-bib-0010] Cotter, S. C. , Simpson, S. J. , Raubenheimer, D. , & Wilson, K. (2011). Macronutrient balance mediates trade‐offs between immune function and life history traits. Functional Ecology, 25, 186–198.

[ece310640-bib-0011] Crone, M. K. , Biddinger, D. J. , & Grozinger, C. M. (2022). Wild bee nutritional ecology: Integrative strategies to assess foraging preferences and nutritional requirements. Frontiers in Sustainable Food Systems, 6, 847003.

[ece310640-bib-0012] Crone, M. K. , & Grozinger, C. M. (2021). Pollen protein and lipid content influence resilience to insecticides in honey bees (*Apis mellifera*). Journal of Experimental Biology, 224, jeb242040. 10.1242/jeb.242040 33758024

[ece310640-bib-0013] DeGrandi‐Hoffman, G. , Gage, S. L. , Corby‐Harris, V. , Carroll, M. , Chambers, M. , Graham, H. , Watkins deJong, E. , Hidalgo, G. , Calle, S. , Azzouz‐Olden, F. , Meador, C. , Snyder, L. , & Ziolkowski, N. (2018). Connecting the nutrient composition of seasonal pollens with changing nutritional needs of honey bee (*Apis mellifera* L.) colonies. Journal of Insect Physiology, 109, 114–124. 29990468 10.1016/j.jinsphys.2018.07.002

[ece310640-bib-0014] Dormann, C. F. , Frund, J. , Bluthgen, N. , & Gruber, B. (2009). Indices, graphs and null models: Analyzing bipartite ecological networks. The Open Ecology Journal, 2, 7–24.

[ece310640-bib-0015] Eckhardt, M. , Haider, M. , Dorn, S. , & Müller, A. (2014). Pollen mixing in pollen generalist solitary bees: A possible strategy to complement or mitigate unfavourable pollen properties? The Journal of Animal Ecology, 83, 588–597. 24164651 10.1111/1365-2656.12168

[ece310640-bib-0016] Filipiak, Z. M. , Denisow, B. , Stawiarz, E. , & Filipiak, M. (2022). Unravelling the dependence of a wild bee on floral diversity and composition using a feeding experiment. Science of the Total Environment, 820, 153326. 35074369 10.1016/j.scitotenv.2022.153326

[ece310640-bib-0017] Filipiak, Z. M. , & Filipiak, M. (2020). The scarcity of specific nutrients in wild bee larval food negatively influences certain life history traits. Biology, 9, 462. 33322450 10.3390/biology9120462 PMC7764569

[ece310640-bib-0018] Fülöp, A. , & Menzel, R. (2000). Risk‐indifferent foraging behaviour in honeybees. Animal Behaviour, 60, 657–666. 11082236 10.1006/anbe.2000.1492

[ece310640-bib-0019] Gaiarsa, M. P. , Rehan, S. , Barbour, M. A. , & McFrederick, Q. S. (2022). Individual dietary specialization in a generalist bee varies across populations but has no effect on the richness of associated microbial communities. The American Naturalist, 200, 730–737. 10.1086/721023 36260853

[ece310640-bib-0020] Haider, M. , Dorn, S. , Sedivy, C. , & Müller, A. (2014). Phylogeny and floral hosts of a predominantly pollen generalist group of mason bees (Megachilidae: Osmiini): Phylogeny and floral hosts of *Osmia* . Biological Journal of the Linnean Society, 111, 78–91.

[ece310640-bib-0021] Jensen, A. B. , Aronstein, K. , Flores, J. M. , Vojvodic, S. , Palacio, M. A. , & Spivak, M. (2013). Standard methods for fungal brood disease research. Journal of Apicultural Research, 52, 1–20. 10.3896/IBRA.1.52.1.13 PMC3816652 24198438

[ece310640-bib-0022] Jones, E. I. , & Dornhaus, A. (2011). Predation risk makes bees reject rewarding flowers and reduce foraging activity. Behavioral Ecology and Sociobiology, 65, 1505–1511.

[ece310640-bib-0023] Kans, J. (2023). Entrez direct: E‐utilities on the unix command line. National Center for Biotechnology Information (US).

[ece310640-bib-0024] Klečka, J. , Mikát, M. , Koloušková, P. , Hadrava, J. , & Straka, J. (2022). Individual‐level specialisation and interspecific resource partitioning in bees revealed by pollen DNA metabarcoding. PeerJ, 10, e13671. 35959478 10.7717/peerj.13671 PMC9359135

[ece310640-bib-0025] Lau, P. , Lesne, P. , Grebenok, R. J. , Rangel, J. , & Behmer, S. T. (2022). Assessing pollen nutrient content: A unifying approach for the study of bee nutritional ecology. Philosophical Transactions of the Royal Society B, 377, 20210510. 10.1098/rstb.2021.0510 PMC9058549 35491590

[ece310640-bib-0026] Leach, M. E. , & Drummond, F. (2018). A review of native wild bee nutritional health. International Journal of Ecology, 2018, 1–10.

[ece310640-bib-0027] LeBuhn, G. , & Vargas Luna, J. (2021). Pollinator decline: What do we know about the drivers of solitary bee declines? Current Opinion in Insect Science, 46, 106–111. 34082166 10.1016/j.cois.2021.05.004

[ece310640-bib-0028] Lucas, A. , Bodger, O. , Brosi, B. J. , Ford, C. R. , Forman, D. W. , Greig, C. , Hegarty, M. , Neyland, P. J. , & De Vere, N. (2018). Generalisation and specialization in hoverfly (Syrphidae) grassland pollen transport networks revealed by DNA metabarcoding. The Journal of Animal Ecology, 87, 1008–1021. 29658115 10.1111/1365-2656.12828 PMC6032873

[ece310640-bib-0029] Machovsky‐Capuska, G. E. , Senior, A. M. , Simpson, S. J. , & Raubenheimer, D. (2016). The multidimensional nutritional niche. Trends in Ecology & Evolution, 31, 355–365. 26993666 10.1016/j.tree.2016.02.009

[ece310640-bib-0030] Mader, E. , Spivak, M. , & Evans, E. (2010). Managing alternative pollinators. Sustainable Agriculture Research and Education.

[ece310640-bib-0031] Michener, C. D. (2007). The bees of the world (2nd ed.). Johns Hopkins University Press.

[ece310640-bib-0032] Nagamitsu, T. , Suzuki, M. F. , Mine, S. , Taki, H. , Shuri, K. , Kikuchi, S. , & Masaki, T. (2018). Effects of forest loss and fragmentation on pollen diets and provision mass of the mason bee, *Osmia cornifrons*, in Central Japan: Forest landscape effects on bee foraging. Ecological Entomology, 43, 245–254.

[ece310640-bib-0033] Parreño, M. A. , Alaux, C. , Brunet, J.‐L. , Buydens, L. , Filipiak, M. , Henry, M. , Keller, A. , Klein, A.‐M. , Kuhlmann, M. , Leroy, C. , Meeus, I. , Palmer‐Young, E. , Piot, N. , Requier, F. , Ruedenauer, F. , Smagghe, G. , Stevenson, P. C. , & Leonhardt, S. D. (2022). Critical links between biodiversity and health in wild bee conservation. Trends in Ecology & Evolution, 37, 309–321. 34955328 10.1016/j.tree.2021.11.013

[ece310640-bib-0034] Phan, N. T. , Joshi, N. K. , Rajotte, E. G. , López‐Uribe, M. M. , Zhu, F. , & Biddinger, D. J. (2020). A new ingestion bioassay protocol for assessing pesticide toxicity to the adult Japanese orchard bee (*Osmia cornifrons*). Scientific Reports, 10, 9517. 32528143 10.1038/s41598-020-66118-2 PMC7289847

[ece310640-bib-0035] Prado, A. , Barret, M. , Vaissière, B. E. , & Torres‐Cortes, G. (2022). Honey bees change the microbiota of pollen. Botanical Sciences, 101, 127–133.

[ece310640-bib-0036] Rau, P. (1937). The Life‐History of Osmia Lignaria and O. Cordata, with Notes on O. Conjuncta1. Annals of the Entomological Society of America, 30(2), 324–343. 10.1093/aesa/30.2.324

[ece310640-bib-0037] R Core Team . (2021). R: A language and environment for statistical computing. R Foundation for Statistical Computing. https://www.R‐project.org/

[ece310640-bib-0038] Rognes, T. , Flouri, T. , Nichols, B. , Quince, C. , & Mahé, F. (2016). VSEARCH: A versatile open source tool for metagenomics. PeerJ, 4, e2584. 27781170 10.7717/peerj.2584 PMC5075697

[ece310640-bib-0039] Ruedenauer, F. A. , Raubenheimer, D. , Kessner‐Beierlein, D. , Grund‐Mueller, N. , Noack, L. , Spaethe, J. , & Leonhardt, S. D. (2020). Best be(E) on low fat: Linking nutrient perception, regulation and fitness. Ecology Letters, 23, 545–554. 31943632 10.1111/ele.13454

[ece310640-bib-0040] Ruedenauer, F. A. , Spaethe, J. , van der Kooi, C. J. , & Leonhardt, S. D. (2019). Pollinator or pedigree: Which factors determine the evolution of pollen nutrients? Oecologia, 191, 349–358. 31463783 10.1007/s00442-019-04494-x

[ece310640-bib-0041] Russo, L. , & Danforth, B. (2017). Pollen preferences among the bee species visiting apple (*Malus pumila*) in New York. Apidologie, 48, 806–820.

[ece310640-bib-0042] Smith, C. , Weinman, L. , Gibbs, J. , & Winfree, R. (2019). Specialist foragers in forest bee communities are small, social or emerge early. Journal of Animal Ecology, 88, 1158–1167. 31063228 10.1111/1365-2656.13003

[ece310640-bib-0043] Sponsler, D. B. , Shump, D. , Richardson, R. T. , & Grozinger, C. M. (2020). Characterizing the floral resources of a north American metropolis using a honey bee foraging assay. Ecosphere, 11, e03102.

[ece310640-bib-0044] Steffan, S. A. , Dharampal, P. S. , Danforth, B. N. , Gaines‐Day, H. R. , Takizawa, Y. , & Chikaraishi, Y. (2019). Omnivory in bees: Elevated trophic positions among all major bee families. The American Naturalist, 194, 414–421. 10.1086/704281 31553217

[ece310640-bib-0045] Tan, K. , Latty, T. , Dong, S. , Liu, X. , Wang, C. , & Oldroyd, B. P. (2015). Individual honey bee (*Apis cerana*) foragers adjust their fuel load to match variability in forage reward. Scientific Reports, 5, 16418. 26549746 10.1038/srep16418 PMC4637910

[ece310640-bib-0046] Thorp, R. W. (1979). Structural, behavioral, and physiological adaptations of bees (Apoidea) for collecting pollen. Annals of the Missouri Botanical Garden, 66, 788.

[ece310640-bib-0047] Vaudo, A. D. , Biddinger, D. J. , Sickel, W. , Keller, A. , & López‐Uribe, M. M. (2020). Introduced bees (*Osmia cornifrons*) collect pollen from both coevolved and novel host‐plant species within their family‐level phylogenetic preferences. Royal Society Open Science, 7, 200225. 32874623 10.1098/rsos.200225 PMC7428236

[ece310640-bib-0048] Vaudo, A. D. , Patch, H. M. , Mortensen, D. A. , Tooker, J. F. , & Grozinger, C. M. (2016). Macronutrient ratios in pollen shape bumble bee (*Bombus impatiens*) foraging strategies and floral preferences. Proceedings of the National Academy of Sciences of the United States of America, 113, E4035–E4042. 27357683 10.1073/pnas.1606101113 PMC4948365

[ece310640-bib-0049] Vaudo, A. D. , Tooker, J. F. , Patch, H. M. , Biddinger, D. J. , Coccia, M. , Crone, M. K. , Fiely, M. , Francis, J. S. , Hines, H. M. , Hodges, M. , Jackson, S. W. , Michez, D. , Mu, J. , Russo, L. , Safari, M. , Treanore, E. D. , Vanderplanck, M. , Yip, E. , Leonard, A. S. , & Grozinger, C. M. (2020). Pollen protein: Lipid macronutrient ratios may guide broad patterns of bee species floral preferences. Insects, 11, 132. 32085627 10.3390/insects11020132 PMC7074338

[ece310640-bib-0050] Vizentin‐Bugoni, J. , Maruyama, P. K. , De Souza, C. S. , Ollerton, J. , Rech, A. R. , & Sazima, M. (2018). Plant‐pollinator networks in the tropics: A review. In W. Dáttilo & V. Rico‐Gray (Eds.), Ecological networks in the tropics (pp. 73–91). Springer International Publishing.

[ece310640-bib-0051] Wickham, H. , François, R. , Henry, L. , & Müller, K. (2022). dplyr: A grammar of data manipulation . https://dplyr.tidyverse.org, https://github.com/tidyverse/dplyr

[ece310640-bib-0052] Williams, N. M. (2003). Consistent mixing of near and distant resources in foraging bouts by the solitary mason bee Osmia lignaria. Behavioral Ecology, 14(1), 141–149. 10.1093/beheco/14.1.141

[ece310640-bib-0053] Zattara, E. E. , & Aizen, M. A. (2021). Worldwide occurrence records suggest a global decline in bee species richness. One Earth, 4, 114–123.

