# Supplementary material for: More than mesolectic: Characterizing the nutritional niche of Osmia cornifrons
Source: Ecol Evol. 2023 Oct 20;13(10):e10640. doi: 10.1002/ece3.10640 (PMC10589078; doi:10.1002/ece3.10640)
Supplement: Supplementary file 1 — Data S1 [file ECE3-13-e10640-s001.docx]

**Supplemental Tables and Figures**

**Table S1. A description of sites and their locations for pollen P:L ratio composition analysis across sites (sites A-D), time (site E), and species composition (site F) in experiments 1A and 1B.**

| **Site** | **Site Type** | **Latitude and longitude** |
| --- | --- | --- |
| A | Orchard | 39.937728, -77.258061 |
| B | Suburban | 39.922389, -77.291664 |
| C | Orchard | 39.937283, -77.258644 |
| D | Orchard | 39.942797, -77.258644 |
| E (The Penn State Arboretum) | Suburban | 40.806599, -77.873160 |
| F (State Game Lands #176) | Seminatural | 40.835440, -77.885504 |
| G (Rock Springs Exp. Farm) | Orchard | 40.708417, -77.953324 |

**Table S2. Recipes for altered diet trials in 2020.** Dry material for each provision measured 220 mg, which was the mean mass of provisions collected from the Arboretum at Penn State in 2019 (data not provided).

| **Diet name** | **P:L ratio** | **Recipe** |
| --- | --- | --- |
| Low P:L diet | 0.4:1 | 220 mg pollen + 90 mg oil + 100 µl sucrose |
| Mid-range P:L diet | 6.6:1 | 220 mg pollen + 8.14 mg protein + 100 µl sucrose |
| High P:L diet | 14.5:1 | 220 mg pollen + 67.54 mg protein +100 µl sucrose |
| Modified control * | 5.5:1 | 220 mg pollen + 30 mg protein +  5.54 mg oil + 100 µl sucrose |
| Control (unaltered pollen) | 5.5:1 | 220 mg pollen + 100 µl sucrose |

 * Modified by adding protein and lipids to change the nutrient concentrations without impacting the overall P:L ratio.

**Table S3. Effect of site and time on *Osmia*-collected larval provisions.** All pairwise comparisons for pollen nutritional composition over time and across sites.

| **By site** | Site name and sample size | A  *n* = 9 | B  *n* = 9 | C  *n* = 9 | D  *n* = 8 | **Protein** | |
| --- | --- | --- | --- | --- | --- | --- | --- |
|  | A |  | 0.0718 | 0.8732 | 0.8758 |  |  |
|  | B |  |  | 0.2959 | 0.3295 |  |  |
|  | C |  |  |  | 0.9999 |  |  |
| **By week (one site)** | Week number and sample size | 1  *n* = 10 | 2  *n* = 9 | 3  *n* = 10 | 4  *n* = 8 | 5  *n* = 9 | 6  *n* = 8 |
|  | 1 |  | 0.8598 | **0.0459** | 0.9840 | 0.9999 | 0.4154 |
|  | 2 |  |  | 0.4900 | 0.9981 | 0.8943 | 0.9711 |
|  | 3 |  |  |  | 0.2769 | 0.0641 | 0.9363 |
|  | 4 |  |  |  |  | 0.9902 | 0.8523 |
|  | 5 |  |  |  |  |  | 0.4757 |

Week df = 5, F = 2.62, Pr(>F) = 0.035; Site df = 3, F = 2.334, Pr(>F) = 0.0932

| **By site** | Site name and sample size | A  *n* = 9 | B  *n* = 9 | C  *n* = 9 | D  *n* = 8 | **Lipids** | |
| --- | --- | --- | --- | --- | --- | --- | --- |
|  | A |  | 0.6086 | 0.9928 | 0.9430 |  |  |
|  | B |  |  | 0.4447 | 0.3112 |  |  |
|  | C |  |  |  | 0.9907 |  |  |
| **By week** | Week number and sample size | 1  *n* = 10 | 2  *n* = 9 | 3  *n* = 10 | 4  *n* = 8 | 5  *n* = 9 | 6  *n* = 8 |
|  | 1 |  | 0.9999 | 0.9999 | 0.8285 | 0.9961 | 0.0769 |
|  | 2 |  |  | 0.9999 | 0.7512 | 0.9996 | 0.0596 |
|  | 3 |  |  |  | 0.7782 | 0.9986 | 0.0612 |
|  | 4 |  |  |  |  | 0.5693 | 0.6696 |
|  | 5 |  |  |  |  |  | **0.0285** |

Week df = 5, F = 2.91, Pr(>F) = 0.0225; Site df = 3, F = 1.232, Pr(>F) = 0.315

| **By site** | Site name and sample size | A  *n* = 9 | B  *n* = 9 | C  *n* = 9 | D  *n* = 8 | **P:L** | |
| --- | --- | --- | --- | --- | --- | --- | --- |
|  | A |  | 0.7857 | 0.7168 | 0.5622 |  |  |
|  | B |  |  | 0.9993 | 0.9772 |  |  |
|  | C |  |  |  | 0.9917 |  |  |
| **By week** | Week number and sample size | 1  *n* = 10 | 2  *n* = 9 | 3  *n* = 10 | 4  *n* = 8 | 5  *n* = 9 | 6  *n* = 8 |
|  | 1 |  | 0.9963 | 0.1433 | 0.7172 | 0.2941 | 0.0713 |
|  | 2 |  |  | 0.3866 | 0.9394 | 0.1318 | 0.2179 |
|  | 3 |  |  |  | 0.9326 | **0.0004** | 0.9972 |
|  | 4 |  |  |  |  | 0.0167 | 0.7659 |
|  | 5 |  |  |  |  |  | **0.0002** |

Week df = 5, F = 6.33, Pr(>F) = 0.0001; Site df = 3, F = 0.663, Pr(>F) = 0.581

**Table S4. Pairwise comparisons of larval survival between treatment groups fed altered pollen diets.** Significant differences are bolded.

| **Larval survival** | Diet | Control modified (5.5:1) | Low (0.4:1) | Mid-range (6.6:1) | High (14.5:1)  *n* = 35 |
| --- | --- | --- | --- | --- | --- |
|  | Unaltered control (5.5:1)  *n* = 45 | **0.0171** | 1 | 0.3971 | **< 0.001** |
|  | Control modified (5.5:1)  *n* = 45 |  | 1 | 0.4537 | 0.5711 |
|  | Low (0.4:1)  *n* = 44 |  |  | 1 | 1 |
|  | Mid-range (6.6:1)  *n* = 49 |  |  |  | **0.0247** |
| **Adult survival** | Diet | Control modified (5.5:1) | Low (0.4:1) | Mid-range (6.6:1) | High (14.5:1) |
|  | Unaltered control (5.5:1) | **<0.001** | - | 0.7295 | 0.9696 |
|  | Control modified (5.5:1) |  | - | **<0.001** | **0.0013** |
|  | Low (0.4:1) |  |  | - | - |
|  | Mid-range (6.6:1) |  |  |  | 0.9834 |

**Table S5. Pairwise comparisons for larval survival and pupation.** Multifloral and monofloral diets were compared to the *Osmia* pollen control group separately. Significant differences are bolded.

| Larval survival | Multifloral diets | 1:1 | 2.9:1 | 5.2:1 | 10.5:1 |  |
| --- | --- | --- | --- | --- | --- | --- |
|  | *Osmia* pollen control | 0.1707 | **0.0181** | 0.5408 | 0.9970 |  |
|  | 1:1 |  | 0.7895 | **0.0097** | 0.3051 |  |
|  | 2.9:1 |  |  | **< 0.001** | **0.0391** |  |
|  | 5.2:1 |  |  |  | 0.3649 |  |
|  | Monofloral diets | *Juglans nigra* (0.6:1) | *Plantago lanceolata* (1.4:1) | *Salix nigra* (2.5:1) | *Dactylis glomerata* (4.2:1) | *Acer rubrum* (6.3:1) |
|  | *Osmia* pollen control | **0.0091** | **0.0104** | 1 | 1 | 0.9988 |
|  | *Juglans nigra* (0.6:1) |  | 0.9877 | 1 | 1 | **0.0042** |
|  | *Plantago lanceolata* (1.4:1) |  |  | 1 | 1 | **0.0051** |
|  | *Salix nigra* (2.5:1) |  |  |  | 1 | 1 |
| Pupation | Multifloral diets | 1:1 | 2.9:1 | 5.2:1 | 10.5:1 |  |
|  | *Osmia* pollen control | **0.0862** | **0.0098** | 0.7897 | 0.9970 |  |
|  | 1:1 |  | 0.7371 | **0.0080** | 0.1681 |  |
|  | 2.9:1 |  |  | **< 0.001** | **0.0204** |  |
|  | 5.2:1 |  |  |  | 0.5981 |  |
|  | Monofloral diets | *Juglans nigra* (0.6:1) | *Plantago lanceolata* (1.4:1) | *Salix nigra* (2.5:1) | *Dactylis glomerata* (4.2:1) | *Acer rubrum* (6.3:1) |
|  | *Osmia* pollen control | **0.0086** | 1 | 1 | 1 | 0.9981 |
|  | *Juglans nigra* (0.6:1) |  | 1 | 1 | 1 | **0.0039** |
|  | *Plantago lanceolata* (1.4:1) |  |  | 1 | 1 | 1 |
|  | *Salix nigra* (2.5:1) |  |  |  | 1 | 1 |

**
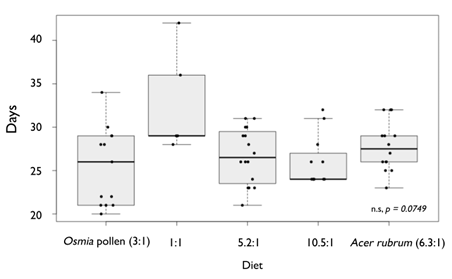
**

**Figure S1. Number of days to cocoon completion.** Diets with fewer than 5 completed cocoons were excluded from this analysis.

**
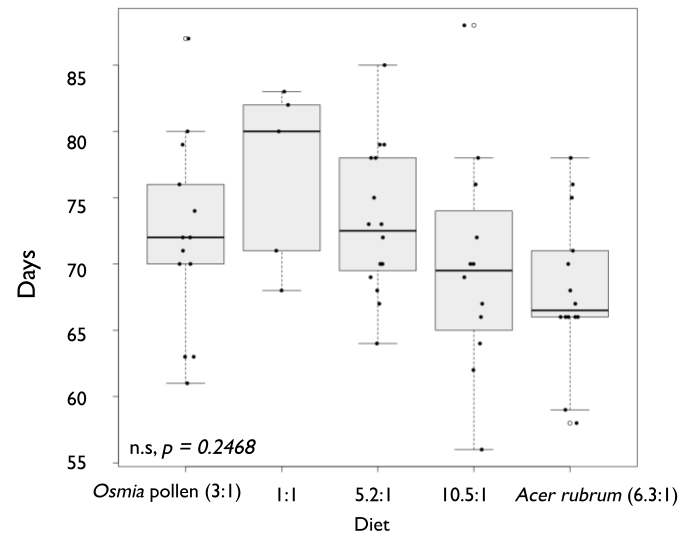
**

**Figure S2. Number of days to pupation.** Diets with fewer than 5 completed cocoons were excluded from this analysis.
